# Exploring plant polyphenols as anti-allergic functional products to manage the growing incidence of food allergy

**DOI:** 10.3389/fnut.2023.1102225

**Published:** 2023-06-09

**Authors:** Tianxiang Wu, Zhenxing Li, Yanyan Wu, Xianqing Yang, Laihao Li, Shengjun Chen, Bo Qi, Yueqi Wang, Chunsheng Li, Yongqiang Zhao

**Affiliations:** ^1^Key Laboratory of Aquatic Product Processing, Ministry of Agriculture and Rural Affairs, National R&D Center for Aquatic Product Processing, South China Sea Fisheries Research Institute, Chinese Academy of Fishery Sciences, Guangzhou, China; ^2^Food Safety Laboratory, Ocean University of China, Qingdao, China; ^3^Co-Innovation Center of Jiangsu Marine Bio-industry Technology, Jiangsu Ocean University, Lianyungang, China; ^4^Collaborative Innovation Center of Seafood Deep Processing, Dalian Polytechnic University, Dalian, China

**Keywords:** anti-allergic, allergen, plant polyphenols, active substances, mechanism, immune activity

## Abstract

The active substances derived from plants have received increasing attention owing to their wide range of pharmacological applications, including anti-tumor, anti-allergic, anti-viral, and anti-oxidative activities. The allergy epidemic is a growing global public health problem that threatens human health and safety. Polyphenols from plants have significant anti-allergic effects and are an important source of anti-allergic drug research and development. Here, we describe recent advances in the anti-allergic efficacy of plant polyphenols, including their comprehensive effects on cellular or animal models. The current issues and directions for future development in this field are discussed to provide a theoretical basis for the development and utilization of these active substances as anti-allergic products.

## 1. Introduction

**FIGURE 1:**
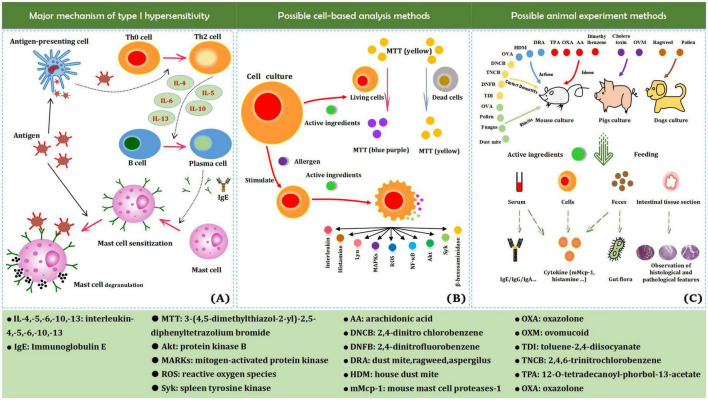
Major mechanism of type I hypersensitivity **(A)**, possible cell-based analysis methods **(B)**, and animal experiment methods **(C)**.

Allergies are pathological immune responses caused by ingestion of, inhalation of, and contact with harmful foreign substances and are aimed at removing the foreign substances (1, 2). Despite their significance to the body’s defense system, their excessiveness (hypersensitivity reaction) is potentially harmful. Hypersensitivity is divided into type I (immediate hypersensitivity), type II (cytotoxic type of allergy), type III (immune complex type), and type IV (delayed type) reactions (3). An allergy is a type of immediate hypersensitivity reaction (abnormal and excessive immune response), characterized by rapid occurrence and rapid regression of the reaction, leading to systemic or local dysfunction. Common allergy symptoms include hives, asthma, rhinitis, and diarrhea. Substances that cause allergic reactions are called allergens. Allergens are mainly macromolecular substances (4). Common allergens originate from foods (e.g., nuts, milk, eggs, soybeans, wheat, peanuts, fish, and crustaceans), inhalants (e.g., pollen, dust, and mites), and microorganisms (e.g., mold and bacteria). Certain physical factors, such as light, can also cause allergies (5). Upon entry into the body, macromolecular substances directly act as antigens, whereas small-molecular substances act as haptens that combine with substances in the body to form new antigens. Food allergens trigger the most allergic diseases, and 90% of allergic diseases are food allergies (6). Upon initial exposure to allergens, the body triggers a series of reaction processes but does not exhibit allergic symptoms. However, it rapidly develops allergic symptoms upon subsequent exposure (Figure 1A). The use of anti-allergic drugs is currently a common solution for the treatment of allergic diseases, but there are still shortcomings such as adverse reactions and drug resistance. Due to the diversity of plant-derived ingredients and their various effects, they are widely used for food and medicine, and are associated with adequate research and utilization prospects (7). This review summarizes research progress in the field of bioactive polyphenols derived from plants with the aim of providing a reference for the development of anti-allergic functional products.

## 2. Anti-allergic effects of plant polyphenols

Polyphenols, including flavonoids, tannins, phenolic acids, and anthocyanins, are important secondary metabolites that are widely present in plants. Further, they can regulate the activities of most enzymes and cell receptors and have significant anti-allergic effects (8). Plant polyphenols interfere with allergic responses mainly by inhibiting the production of signaling factors and suppressing cytokine production, signal transduction, and gene expression in mast cells, basophils, or T-cells (Table 1). The binding between allergenic proteins and plant polyphenols changes the conformation of the protein, reducing its allergenic potential (9, 10). The activity of polyphenols in inhibiting allergic reactions is related to their structure. Among the many components of epigallocatechin gallate, the component with a pyrogallol structure, a galloyl group on the benzene ring, and a 2,3-*cis* configuration has the strongest inhibitory activity (11). Functional groups that enhance inhibitory activity often have a synergistic and therefore stronger effect.

**TABLE 1:** Origins and anti-allergic mechanisms of plant polyphenols.

Name	Source	Allergic reaction model	Anti-allergic mechanism
Baicalin	*Scutellaria baicalensis Georgi*	PBMCs C-m; OVA-S M-m	Inhibits autophagy to regulate Th17/Treg cell differentiation and the release of IgE, histamine, IL-1β, IL-4, IL-6 and TNF-α↓ (17, 26).
Hydroxysafflor yellow A	Safflower (*Carthamus tinctorius* L.)	Guinea pig asthma model; hindpaw extravasation M-m	Th1/Th2 cell imbalance↑ and activation of the MAPK signaling pathway↓(27). Intracellular Ca^2+^ mobilization↓ and the release of cytokines from mast cells↓ (28).
Luteolin	Perilla (*Perilla frutescens*)	OVA-S M-m; Dust mite sensitization M-m; PBMCs C-m; RBL-2H3 C-m	Regulates the TLR4/NF-κB pathway (29). Activates the PI3K/Akt/mTOR pathway, inhibits Beclin-1-PI3KC3 complex and IL-4 (18, 30). Expression of GATA-3 ⇓ and IFN-γ in dendritic cells ⇑ (15).
Quercetin	Mukdenia (*Mukdenia rossii (Oliv.) Koidz.*)	OVA-S asthma M-m; Conjunctivitis M-m; OVA sensitized M-m	IgE, TNF-α, IL-1β, IL-4, and IL-5 cytokines↓ and IFN-π↑ (31). Lyn/PLC gamma/IP3R-Ca^2+^, Lyn/ERK1/2, and Lyn/NF-κB signaling↓, expression of histamine H-1 receptor gene↓ (16, 32). Modulates the equilibrium drift of Th1/Th2 cells (33).
Isoquercitrin	Hops (*Humulus lupulus* L.); onion (*Allium cepa*)	OVA-S rhinitis M-m; RBL-2H3 C-m	Mast cell release of histamine↓, intracellular calcium influx/consumption↓, and Th1/Th2 cell imbalance ⇑ (19, 34).
Kaempferol	Kencur (*Kaempferol galanga* L.)	C48/80-induced PCA model; OVA-S Guinea pig model	PLC γ phosphorylation to Ca^2+^ mobilization↓, IL-5, IL-13, and GM-CSF↓ (35, 36).
Epigallocatechin gallate	Tea leaves (*Camellia sinensis* (L.) *O. Kuntze*)	Nc/Nga M-m of AD	Serum IgE, histamine levels, IFN-γ, and Th2-type cytokines↓ (37).
Chestnut inner shell extract	chestnut; (*Castanea crenata*)	OVA-S asthma M-m	Phosphorylation of NF-κB↓ and expression of metalloproteinase-9↓ (38).
Phlorotannins	Brown alga (*Ecklonia* sp.)	RBL-2H3 C-m; Allergic inflammation M-m	Mast cell degranulation↓ and Ca^2+^ influx↓. Expression of FcεRI on the cell surface ↓, and the expression of MAPK and NF-κB signaling pathways↓ (39).
Rosmarinic acid	Rosemary (*Rosmarinus officinalis*)	OVA-S M-m	Production of IgE, IL-4, IL-5, and IL-13↓; secretion of IFN-γ↑. ROS production↓ and the activities of SOD↑, glutathione peroxidase↑, catalase↑. Cu/Zn SOD expression in lung tissue↑, NOX-2 and NOX-4 expression↓ (40, 41).

OVA-S, ovalbumin-sensitized; C-m, cell model; M-m, mouse model; PBMCs, peripheral blood mononuclear cells; MAPK, mitogen-activated protein kinase; ERK1/2, extracellular regulated kinases 1/2; ⇓, ⇑, in vitro; ↓, ↑, in vivo.

Flavonoids are the polyphenols associated with the most outstanding research progress in recent years. They mainly affect the secretion process of immune cells, mitosis, and the interaction between cells and significantly influence the inhibition of allergic reactions (12). Flavonoids inhibit the release of the proteolytic enzyme β-hexosaminidase and histamine from mast cells (13, 14). Certain flavonoids can modulate the expression of genes associated with allergic reactions. Luteolin can promote the expression of IFN-γ in dendritic cells, and quercetin inhibits the transcriptional upregulation of histamine H-1 receptors by inhibiting the protein kinase C-δ/extracellular signal-regulated kinase/poly (ADP-ribose) polymerase-1 signaling pathway mediated transcriptional upregulation of receptors (15, 16). Baicalin, luteolin, isoquercitrin, and other flavonoids can inhibit the release of cell mediators from mast cells, reduce the expression level of Th2 cell molecules, improve the balance between Th1 cells and Th2 cells, and inhibit the release of related enzymes and gene expression levels, thereby exerting anti-allergic effects (17–19).

Flavonoids have a C6-C3-C6 skeleton, two benzene rings, and a heterocyclic ring in the middle (20). Flavonoids with hydroxyl groups on the benzene ring or carbon–carbon double bonds on the heterocycle have more significant anti-allergic effects, and the enhancing effects of hydroxyl groups and carbon–carbon double bonds can be superimposed. Both luteolin and eriodictyol are flavonoids with very similar structures. Luteolin, with a carbon–carbon double bond on the heterocycle, has a significant anti-allergic effect, whereas eriodictyol, without a carbon–carbon double bond on the heterocycle, has almost no anti-allergic effect (21). Notably, the introduction of a hydroxyl group to the heterocycle has no significant effect on the enhancement of anti-allergic efficacy. The glucuronidation reaction is the main metabolic pathway associated with flavonoids (22). However, flavonoids with significant glucuronidation, such as alpinetin, have poor bioavailability *in vivo* (23). The inhibition of glucuronidation can thus significantly improve the pharmacokinetic profile of flavonoids. In conclusion, flavonoids have significant anti-allergic effects, which are highly correlated with their structure. By modifying the structure of plant-derived flavonoids, their bioavailability and pharmacokinetics *in vivo* can be improved, and their anti-allergic effects can be enhanced. The development of anti-allergic drugs using flavonoids as raw materials is therefore anticipated. Polyphenols have significant anti-allergic activity, but there are fewer studies on their development for the treatment of allergies, probably because of their low bioavailability in the body. Improving the body’s absorption of polyphenols by modifying their structure has interesting prospects and warrants further study.

Several studies have shown that changing the contents of anti-allergic active substances in food can reduce the incidence of food allergy. An epidemiological survey in Finland showed that the incidence of allergic asthma could be reduced by the relatively high intake of flavonoids (24). Another study showed that cocoa intake of more than 7 g per day can reduce the incidence of food allergy, which might be related to the flavonoids contained in this food (25). In conclusion, modifying the anti-allergic active ingredients in food to reduce the incidence of food allergy is feasible.

## 3. *In vitro* and *in vivo* research methods

### 3.1. *In vitro* studies

*In vitro* studies mainly use enzymes and cells as research tools. Hyaluronidase, an enzyme that inhibits the activity of hyaluronic acid, is strongly associated with allergic reactions (42). Hyaluronic acid is a mucopolysaccharide widely distributed in epithelial tissue, which plays an important role in maintaining the physiological function of the skin or mouth. The activation of hyaluronidase will lead to the decomposition of hyaluronic acid, change the permeability of capillaries and inflammatory cells, and induce allergic reactions (43). Certain drugs for the treatment of allergic reactions have strong inhibitory effects on hyaluronidase activity (44); therefore, hyaluronidase is often used to test the anti-allergic activity of active compounds.

In recent years, the use of cell experiments has been integrated into the field of anti-allergic research. Most cell models are associated with similar experimental methods, including the use of MTT to measure cell viability to prove the safety of the test material, as well as the effect of active substances on cell degranulation, the release of histamine, intracellular reactive oxygen species, and the secretion of cytokines (Figure 1B). Mast cells are the main effector cells in allergic diseases, but the use of suitable sub-cultured mast cell lines for research is lacking (45). The high-affinity IgE receptor (FcεRI) is present on the surface of RBL-2H3 cells, which have numerous characteristics and functions of mast cells. More importantly, RBL-2H3 cells can be passaged more stably than mast cells. Therefore, RBL-2H3 cells have been commercialized as an alternative model of mast cells for the study of allergic diseases (46). The protein kinases Fyn, Lyn, and Syk in RBL-2H3 cells are activated in the initial stage of degranulation signaling; besides, Fyn and Lyn can further activate sphingosine kinase, and Syk mediates the degranulation of RBL-2H3 cells via the production of phosphatidylinositol triphosphate (47). Compound 48/80 is commonly used to induce the activation and degranulation of RBL-2H3 cells (48). The release rate of β-hexosaminidase and the amount of histamine released are markers used to detect the degranulation of RBL-2H3 cells (49). However, RBL-2H3 cells are only suitable for studying IgE-mediated degranulation and are not representative of other irritants (Compound 48/80, etc.) (50). Thus, RBL-2H3 cells might not be suitable for establishing anaphylactoid reaction models.

The RBL-2H3 cell model has been used to study the anti-allergic activity of different polyphenol combinations. One study showed that curcumin or quercetin combined with luteolin could significantly inhibit TNF-α and IL-8 mRNA expression. In addition, the combination of luteolin and quercetin inhibits the β- activity of hexosaminidase (51). *Persicaria perfoliata* (L.) H. Gross is commonly used as an anti-allergic drug in traditional medicine. The RBL-2H3 cell model was used to show that an aqueous extract of *P. perfoliata* could inhibit the release of β-hexosaminidase in a dose-dependent manner, without causing cytotoxicity. Treatment with an aqueous extract of *P. perfoliata* also dose-dependently inhibited the phosphorylation of extracellular regulatory kinase (ERK) and c-Jun N-terminal kinase (JNK) in cells (52).

The P815 cell line is a mast cell model. Compared with RBL-2H3 cells, P815 cells have a shorter degranulation time and a more severe response to the same external stimulus, and the percentage and degranulation ratio of Annexin V-positive cells among P815 cells were found to be substantially higher than those in RBL-2H3 cells. The use of P815 cells has shown that stem cell factors have an important effect on cytokine production by mast cells (significantly increased IL-13 production, marked ERK phosphorylation, and CREB activation) (53). The P815 cell model was further used to show that zinc inhibits mast cell hypersensitivity through the IL-33/ST2 pathway (decreased expression of IL-33, ST2, p38, and p65 proteins) (54).

Basophils are a type of white blood cells that express the high-affinity IgE receptor (Fc&RI) on their surface. After basophils come into contact with allergens, they release histamine, leukotriene C4, IL-4, and IL-13 (55). Further, basophils express various markers such as CD45, CD63, and CD203c on their surface (56). Therefore, they are sometimes used in allergy research. When basophils are stimulated, they become activated, the basophilic granule membrane fuses with the cell membrane, and CD63 is exposed (57). Therefore, the degranulation of activated basophils can be detected via flow cytometry. CD203c expression increases when basophils are activated and thus can be used as an indicator of basophil activation through flow cytometry.

In summary, *in vitro* anti-allergic studies are primarily conducted using enzymes and cells. Experiments using enzymes are simple, whereas those using cells provide an in-depth explanation of the mechanism by which active compounds exert anti-allergic effects. At present, the commonly used cell models still need improvements with respect to stability between cell passages and the reproducibility of experimental data, in addition to reducing interference with experimental results owing to cell instability.

### 3.2. *In vivo* studies

*In vivo* studies of allergic reactions mainly use animals as experimental subjects. Animal experiments are widely used in life science research and play an important role therein (58). Guinea pigs, BALB/c mice, Wistar rats, Sprague Dawley rats, Brown Norway rats, piglets, and dogs have been used in experimental anti-allergic studies (59–65). Rodents, such as Guinea pigs, rats, and mice, have short reproductive cycles, are less costly, and can be easily bred, with mature commercial models available, making them more suitable as anti-allergic research animal models (66). Mouse models are mainly used to obtain serum data, excreta data, and tissue section data (Figure 1C).

Mice are the most commonly used animal models, and various mouse models have been developed for anti-allergic experiments, including BALB/c, C57BL/6, and A/J mice. The BALB/c mouse is the most commonly used mouse model and is a highly IgE-responsive strain. Mice sensitized with a high dose of tropomyosin exhibit signs of anaphylaxis, including puffiness around the eyes and snout, inactivity, tremor, and convulsions, after the challenge (67). After the intraperitoneal injection of ovalbumin (OVA), BALB/c mice show mild allergic signs, such as piloerection and rapid breathing, increased serum levels of OVA sIgE, sIgG, HIS, and mMCP-1, and an increase in the percentage of Th2 cells that differentiate among splenic lymphocytes, indicating a Th2-type allergic reaction (68). Some scholars have explored the feasibility of using BALB/c mice as anti-allergic research animal models and found that these mice exhibit different levels of sensitization to various allergens and can be used to distinguish sensitization to different food proteins (69).

In conclusion, *in vivo* experiments on active compounds are mainly conducted on animals. Paradoxically, animals that are similar to humans tend to have long life cycles and high testing costs, and conclusions obtained from animals that are easy to use as experimental subjects are often difficult to reproduce in humans. The direct use of humans as experimental subjects is very dangerous and can lead to unknown consequences. This necessitates the identification of new and more suitable animal models or the improvement of existing animal models to further optimize the efficiency of animal experiments and the accuracy of results. In addition, an increasing number of studies are based on animal models to study the relationship between intestinal microorganisms and food allergy, which is a promising research field.

## 4. Conclusion

Plant polyphenols have remarkable effects and limited toxic and side effects, but at present, plant medicines are not widely used for allergy treatment. The main problems are as follows. First, research still focuses on plant polyphenols that are known to have anti-allergic activity, such as quercetin and paeonol. Second, most anti-allergic activity studies stay at the level of activity characterization and verification; there are few studies on the molecular targeting mechanism and even fewer on their deep application to drug development. Third, there are few studies on the structure–activity relationship of plant polyphenols and the key functional groups and spatial structures that characterize their efficacy. There are also limited studies on plant polyphenols and even fewer toxicological studies on them, all of which require more in-depth research. Finally, plant polyphenols have inherent cross-reactivity with many reported allergens, which severely affects their application. A possible solution to this is to suppress the effects of cross-allergic allergens by mixing various plant polyphenols. Another possible solution is to separate and purify the components of plant polyphenols, separating proteins from other components, thereby reducing the possibility of cross-reactions with other allergens. Conversely, as almost all drugs have side effects, if the inhibitory effect of plant polyphenols on allergic reactions is weaker than the allergic symptoms caused by allergens produced by the plant itself, then the effect is of no value.

In the background of the current allergic disease epidemic sweeping the world and affecting the health and life of numerous humans, the market demand for anti-allergic drugs will increase. At the same time, the continuous introduction of new foods and the continuous refinement of food processing will continue to generate new allergens, and the demand for allergy-preventing foods and drugs that can broadly inhibit allergic reactions is increasing. Currently marketed anti-allergic drugs, such as loteprednol and jasmonic acid, have single effects and marked toxic and side effects, which limit the application of chemically synthetized anti-allergic drugs. In contrast, traditional Chinese medicines have fewer side effects, a wide range of effects, and better applications. However, their compositions are complex, their mechanisms are difficult to demonstrate, and they are difficult to market as drugs. Therefore, in-depth studies on the structure–activity relationship, mechanisms of action, efficacy, toxicology, and clinical application potential of anti-allergic active substances should be carried out to make active substances more attractive drugs. Future research should focus more on improving the bioavailability of natural plant polyphenols for practical applications that suppress the prevalence of food allergies. The development of natural active compounds as food components or pharmaceutical formulation components could be a viable approach.

## Author contributions

TW and ZL: writing—original draft preparation. YZ and YaW: conceptualization. XY: funding acquisition and supervision. YZ, XY, LL, and SC: writing—review and editing. TW, BQ, YuW, and CL: visualization. All authors read and agreed to the published version of the manuscript.
